# 5-Carbamoyl-2-methyl-1-(2-methyl­benz­yl)pyridinium bromide

**DOI:** 10.1107/S1600536812018958

**Published:** 2012-05-05

**Authors:** Kyung Beom Kim, Seung Man Yu, Cheal Kim, Youngmee Kim

**Affiliations:** a Department of Fine Chemistry, Seoul National University of Science & Technology, Seoul 139-743, Republic of Korea; b Department of Chemistry and Nano Science, Ewha Womans University, Seoul 120-750, Republic of Korea

## Abstract

In the title mol­ecular salt, C_15_H_17_N_2_O^+^·Br^−^, the benzene and pyridinium rings form a dihedral angle of 83.0 (1)°. In the crystal, N—H⋯Br and N—H⋯O hydrogen bonds link the components into chains along [010]. These chains are linked by weak C—H⋯O and C—H⋯Br hydrogen bonds, forming a three-dimensional network.

## Related literature

The title compound was prepared as an NAD+ (nicotinamide adenine dinucleotide) model. For the role of reduced nicotinamide co-factors (NADH and NADPH) in enzyme-catalysed reactions, see: Hollmann *et al.* (2001 ▶); Lee *et al.* (2011 ▶); Maenaka *et al.* (2012 ▶); Park *et al.* (2008 ▶); Ruppert *et al.* (1988 ▶); Zhu *et al.* (2003 ▶, 2006 ▶). For the generation of NADH, see: Qing *et al.* (2006 ▶). For an efficient method of *in situ* regeneration, see: Song *et al.* (2008 ▶). For a related structure, see: Kim *et al.* (2012 ▶).
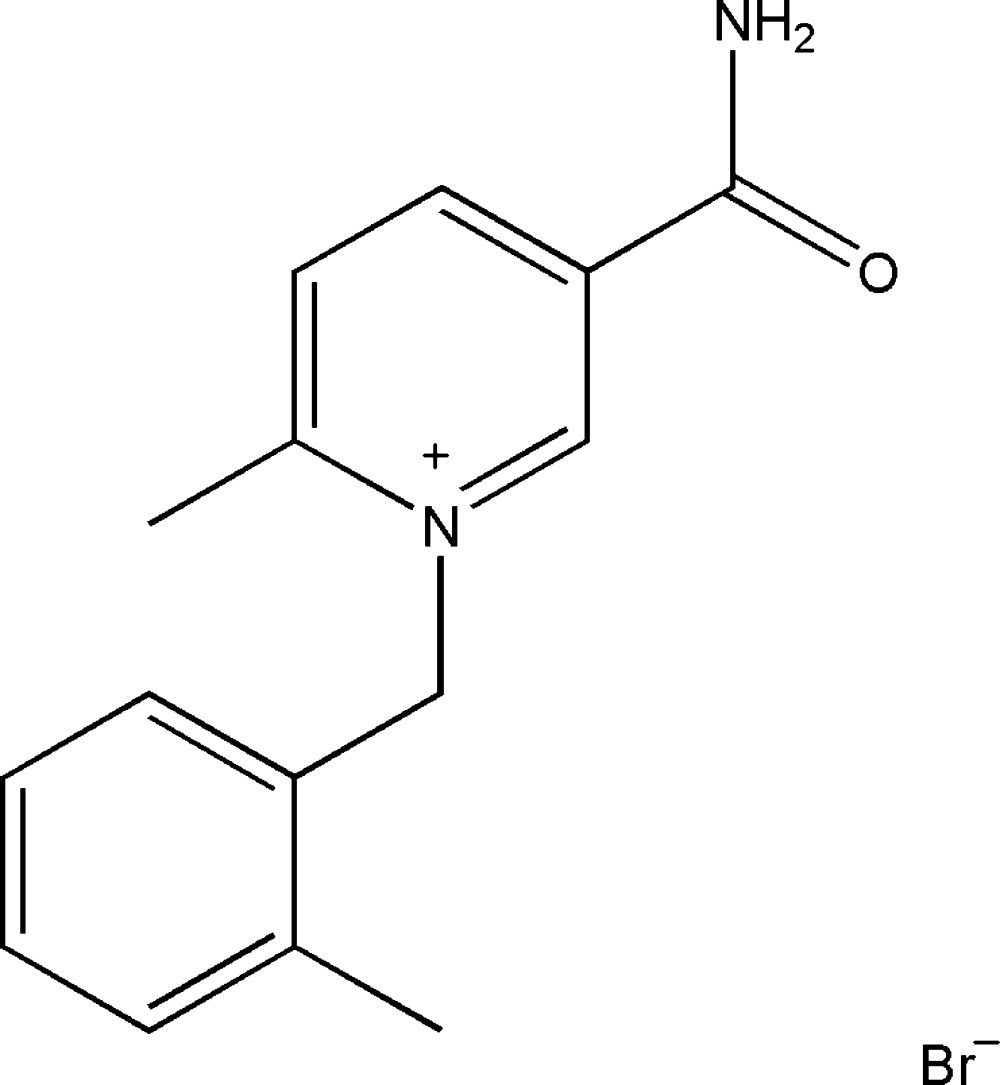


## Experimental

### 

#### Crystal data

C_15_H_17_N_2_O^+^·Br^−^
*M* *_r_* = 321.22
Monoclinic, 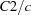

*a* = 8.4880 (17) Å
*b* = 10.502 (2) Å
*c* = 33.450 (7) Å
β = 96.85 (3)°
*V* = 2960.5 (10) Å^3^
*Z* = 8
Mo *K*α radiation
μ = 2.77 mm^−1^
*T* = 293 K
0.50 × 0.20 × 0.10 mm

#### Data collection

Bruker SMART CCD diffractometer
Absorption correction: multi-scan (*SADABS*; Bruker, 1997 ▶) *T*_min_ = 0.338, *T*_max_ = 0.769
8103 measured reflections
2879 independent reflections
1857 reflections with *I* > 2σ(*I*)
*R*_int_ = 0.043

#### Refinement

*R*[*F* ^2^ > 2σ(*F* ^2^)] = 0.042
*wR*(*F* ^2^) = 0.096
*S* = 1.00
2879 reflections
174 parameters
H-atom parameters constrained
Δρ_max_ = 0.36 e Å^−3^
Δρ_min_ = −0.31 e Å^−3^

### 

Data collection: *SMART* (Bruker, 1997 ▶); cell refinement: *SAINT* (Bruker, 1997 ▶); data reduction: *SAINT*; program(s) used to solve structure: *SHELXS97* (Sheldrick, 2008 ▶); program(s) used to refine structure: *SHELXL97* (Sheldrick, 2008 ▶); molecular graphics: *DIAMOND* (Brandenburg, 1999 ▶); software used to prepare material for publication: *SHELXTL* (Brandenburg, 1999 ▶).

## Supplementary Material

Crystal structure: contains datablock(s) I, global. DOI: 10.1107/S1600536812018958/lh5461sup1.cif

Structure factors: contains datablock(s) I. DOI: 10.1107/S1600536812018958/lh5461Isup2.hkl

Supplementary material file. DOI: 10.1107/S1600536812018958/lh5461Isup3.cml

Additional supplementary materials:  crystallographic information; 3D view; checkCIF report

## Figures and Tables

**Table 1:** Hydrogen-bond geometry (Å, °)

*D*—H⋯*A*	*D*—H	H⋯*A*	*D*⋯*A*	*D*—H⋯*A*
N1—H1*A*⋯Br1	0.86	2.67	3.477 (3)	157
N1—H1*B*⋯O1^i^	0.86	2.35	3.207 (4)	173
C3—H3⋯O1^i^	0.93	2.22	3.149 (5)	174
C4—H4⋯Br1^i^	0.93	2.78	3.712 (4)	175
C6—H6*C*⋯Br1^ii^	0.96	2.73	3.638 (3)	157
C8—H8*B*⋯Br1^iii^	0.97	2.87	3.782 (3)	156

Symmetry codes: (i) 
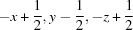
; (ii) 
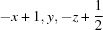
; (iii) 
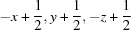
.

**Fig. 1.**
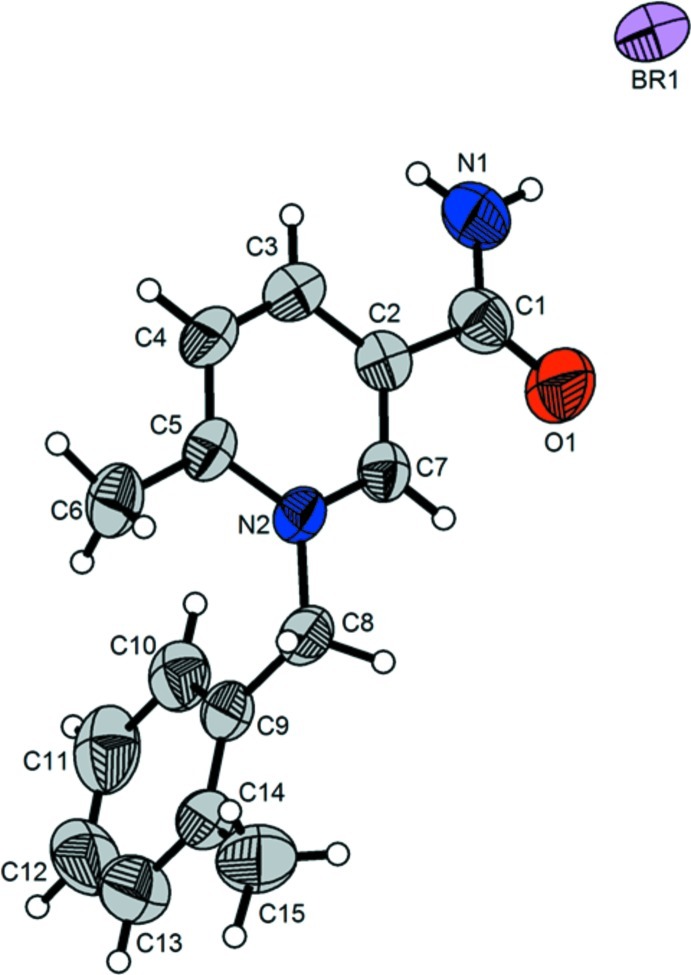
The molecular structure of the title compound showing displacement ellipsoids at the 50% probability level.

## supplementary crystallographic information

### Comment

Reduced nicotinamide co-factors (NADH and NADPH) play an important role in a variety of enzyme-catalyzed reactions (Hollmann *et al.*, 2001; Lee *et al.*, 2011; Maenaka *et al.*, 2012; Park *et al.*, 2008; Ruppert *et al.*, 1988; Zhu *et al.*, 2003; Zhu *et al.*, 2006). For example, in biocatalytic systems, many enzymes depend on nicotinamide co-factors (NAD and NADP) for their functions (Park *et al.*, 2008).To date, a number of strategies including enzymatic catalysis, whole-cell conversion, chemical method, have been devised to the regeneration of NADH (Qing *et al.*, 2006). The high cost of these co-factors, however, is prohibitive of industrialization of many promising enzymatic processes. An efficient method of their *in situ* regeneration is the only means for making the processes economically and industrially feasible (Song *et al.*, 2008). Therefore, we and many researchers have given considerable attention to the chemistry of NADH and its models (Hollmann *et al.*, 2001; Kim *et al.*, 2012). In this work, we have synthesized the title compound as a NAD^+^ model and report here the crystal structure.

The molecular structure of the title compound is shown in Fig. 1. The benzene and pyridinium rings form a dihedral angle of 83.0 (1)°. In the crystal, N—H···Br and N—H···O hydrogen bonds link the components into chains along [010]. These chains are linked by weak intermolecular C—H···O and C—H···Br hydrogen bonds to form a three-dimensional network.

### Experimental

6-Methylnicotinamide (136.15 mg, 1 mmol) was dissolved 10 ml dimethylformamide. After stirring for a few minutes, 2-methylbenzyl bromide (185.06 mg, 1 mmol) was carefully added to the reaction solution. The solution was stirred for 3 h at 353K. The precipitate was filtered, washed three times with methylene chloride, and dried under vacuum. 3-Carbamoyl-6-methyl-1-(2-methylbenzyl)pyridinium bromide (16.06 mg, 0.05 mmol) was dissolved in 1 ml methanol and carefully layered by 3 ml isopropyl ether. Suitable crystals of the title compound for X-ray analysis were obtained in a few days.

### Refinement

H atoms bonded to C atoms were placed in calculated positions with C—H distances of 0.93 Å for aromatic C atoms, 0.96 Å for methyl C atoms, and 0.97 Å for a methylene C atoms. They were included in the refinement in riding-motion approximation with *U*_iso_(H) = 1.2*U*_eq_(C) and 1.5*U*_eq_(C). The positions of N—H atoms of the amine were included with N—H = 0.86 A and *U*iso(H) =1.2*U*_eq_(N).

### Crystal data

**Table:**

C_15_H_17_N_2_O^+^·Br^−^	*F*(000) = 1312
*M**_r_* = 321.22	*D*_x_ = 1.441 Mg m^−^^3^
Monoclinic, *C*2/*c*	Mo *K*α radiation, λ = 0.71073 Å
Hall symbol: -C 2yc	Cell parameters from 2248 reflections
*a* = 8.4880 (17) Å	θ = 2.3–25.2°
*b* = 10.502 (2) Å	µ = 2.77 mm^−^^1^
*c* = 33.450 (7) Å	*T* = 293 K
β = 96.85 (3)°	Block, colorless
*V* = 2960.5 (10) Å^3^	0.50 × 0.20 × 0.10 mm
*Z* = 8	

### Data collection

**Table:**

Bruker SMART CCD diffractometer	2879 independent reflections
Radiation source: fine-focus sealed tube	1857 reflections with *I* > 2σ(*I*)
Graphite monochromator	*R*_int_ = 0.043
φ and ω scans	θ_max_ = 26.0°, θ_min_ = 2.5°
Absorption correction: multi-scan (*SADABS*; Bruker, 1997)	*h* = −9→10
*T*_min_ = 0.338, *T*_max_ = 0.769	*k* = −12→8
8103 measured reflections	*l* = −41→40

### Refinement

**Table:**

Refinement on *F*^2^	Primary atom site location: structure-invariant direct methods
Least-squares matrix: full	Secondary atom site location: difference Fourier map
*R*[*F*^2^ > 2σ(*F*^2^)] = 0.042	Hydrogen site location: inferred from neighbouring sites
*wR*(*F*^2^) = 0.096	H-atom parameters constrained
*S* = 1.00	*w* = 1/[σ^2^(*F*_o_^2^) + (0.0247*P*)^2^ + 1.3939*P*] where *P* = (*F*_o_^2^ + 2*F*_c_^2^)/3
2879 reflections	(Δ/σ)_max_ = 0.001
174 parameters	Δρ_max_ = 0.36 e Å^−^^3^
0 restraints	Δρ_min_ = −0.31 e Å^−^^3^

### Special details

**Table:**

Geometry. All e.s.d.'s (except the e.s.d. in the dihedral angle between two l.s. planes) are estimated using the full covariance matrix. The cell e.s.d.'s are taken into account individually in the estimation of e.s.d.'s in distances, angles and torsion angles; correlations between e.s.d.'s in cell parameters are only used when they are defined by crystal symmetry. An approximate (isotropic) treatment of cell e.s.d.'s is used for estimating e.s.d.'s involving l.s. planes.
Refinement. Refinement of *F*^2^ against ALL reflections. The weighted *R*-factor *wR* and goodness of fit *S* are based on *F*^2^, conventional *R*-factors *R* are based on *F*, with *F* set to zero for negative *F*^2^. The threshold expression of *F*^2^ > σ(*F*^2^) is used only for calculating *R*-factors(gt) *etc*. and is not relevant to the choice of reflections for refinement. *R*-factors based on *F*^2^ are statistically about twice as large as those based on *F*, and *R*- factors based on ALL data will be even larger.

### Fractional atomic coordinates and isotropic or equivalent isotropic displacement parameters (Å^2^)

**Table:**

	*x*	*y*	*z*	*U*_iso_*/*U*_eq_	
Br1	0.01684 (4)	0.38708 (4)	0.349683 (12)	0.06234 (17)	
N1	0.2074 (3)	0.3760 (3)	0.26330 (9)	0.0686 (9)	
H1A	0.1599	0.4033	0.2830	0.082*	
H1B	0.2111	0.2957	0.2585	0.082*	
N2	0.4953 (3)	0.4466 (3)	0.15096 (8)	0.0435 (6)	
O1	0.2735 (4)	0.5725 (3)	0.24558 (9)	0.0862 (9)	
C1	0.2742 (4)	0.4572 (4)	0.24056 (11)	0.0577 (9)	
C2	0.3589 (4)	0.4032 (3)	0.20721 (10)	0.0466 (8)	
C3	0.3794 (4)	0.2738 (4)	0.20086 (11)	0.0590 (9)	
H3	0.3414	0.2144	0.2180	0.071*	
C4	0.4560 (4)	0.2342 (3)	0.16924 (11)	0.0570 (9)	
H4	0.4696	0.1475	0.1652	0.068*	
C5	0.5131 (4)	0.3198 (3)	0.14343 (10)	0.0468 (8)	
C6	0.5919 (4)	0.2774 (3)	0.10809 (10)	0.0599 (10)	
H6A	0.5354	0.3112	0.0838	0.090*	
H6B	0.5916	0.1861	0.1068	0.090*	
H6C	0.6993	0.3078	0.1109	0.090*	
C7	0.4195 (3)	0.4868 (3)	0.18186 (9)	0.0452 (8)	
H7	0.4083	0.5737	0.1860	0.054*	
C8	0.5564 (4)	0.5431 (3)	0.12466 (10)	0.0503 (9)	
H8A	0.6635	0.5199	0.1201	0.060*	
H8B	0.5617	0.6246	0.1384	0.060*	
C9	0.4566 (4)	0.5572 (3)	0.08456 (10)	0.0500 (9)	
C10	0.3025 (4)	0.5121 (3)	0.07804 (11)	0.0609 (10)	
H10	0.2579	0.4712	0.0986	0.073*	
C11	0.2144 (5)	0.5281 (4)	0.04038 (14)	0.0816 (13)	
H11	0.1113	0.4971	0.0356	0.098*	
C12	0.2825 (7)	0.5909 (4)	0.01029 (14)	0.0939 (17)	
H12	0.2248	0.6027	−0.0149	0.113*	
C13	0.4333 (7)	0.6351 (4)	0.01754 (14)	0.0880 (14)	
H13	0.4773	0.6767	−0.0030	0.106*	
C14	0.5231 (5)	0.6206 (3)	0.05406 (12)	0.0664 (11)	
C15	0.6904 (6)	0.6707 (5)	0.06056 (15)	0.1029 (16)	
H15A	0.7139	0.7144	0.0368	0.154*	
H15B	0.7629	0.6010	0.0661	0.154*	
H15C	0.7010	0.7286	0.0829	0.154*	

### Atomic displacement parameters (Å^2^)

**Table:**

	*U*^11^	*U*^22^	*U*^33^	*U*^12^	*U*^13^	*U*^23^
Br1	0.0576 (3)	0.0523 (3)	0.0775 (3)	−0.0027 (2)	0.00950 (18)	0.0134 (2)
N1	0.075 (2)	0.078 (2)	0.0571 (19)	−0.0028 (19)	0.0258 (17)	−0.0019 (17)
N2	0.0468 (15)	0.0348 (15)	0.0494 (16)	0.0015 (13)	0.0076 (13)	−0.0034 (13)
O1	0.118 (2)	0.0592 (18)	0.091 (2)	0.0093 (18)	0.0527 (18)	−0.0127 (16)
C1	0.057 (2)	0.065 (3)	0.052 (2)	0.007 (2)	0.0107 (18)	−0.001 (2)
C2	0.0435 (18)	0.051 (2)	0.0451 (19)	0.0017 (16)	0.0045 (15)	−0.0026 (17)
C3	0.071 (2)	0.045 (2)	0.061 (2)	−0.0051 (19)	0.011 (2)	0.0024 (18)
C4	0.072 (2)	0.035 (2)	0.065 (2)	0.0027 (18)	0.0104 (19)	−0.0033 (18)
C5	0.0427 (19)	0.037 (2)	0.059 (2)	0.0023 (16)	0.0002 (16)	−0.0075 (17)
C6	0.057 (2)	0.053 (2)	0.071 (2)	0.0049 (18)	0.0164 (19)	−0.0177 (19)
C7	0.0461 (19)	0.044 (2)	0.0453 (19)	0.0041 (16)	0.0042 (16)	−0.0068 (16)
C8	0.057 (2)	0.038 (2)	0.058 (2)	−0.0058 (17)	0.0144 (18)	−0.0061 (17)
C9	0.064 (2)	0.0372 (19)	0.050 (2)	0.0100 (18)	0.0107 (18)	−0.0060 (17)
C10	0.065 (2)	0.054 (2)	0.062 (3)	0.018 (2)	0.001 (2)	−0.0070 (19)
C11	0.078 (3)	0.073 (3)	0.090 (3)	0.029 (2)	−0.008 (3)	−0.021 (3)
C12	0.142 (5)	0.079 (4)	0.055 (3)	0.046 (3)	−0.014 (3)	0.002 (2)
C13	0.129 (4)	0.071 (3)	0.064 (3)	0.014 (3)	0.013 (3)	0.004 (2)
C14	0.103 (3)	0.042 (2)	0.055 (2)	0.007 (2)	0.014 (2)	−0.0017 (19)
C15	0.137 (4)	0.088 (3)	0.092 (3)	−0.044 (3)	0.048 (3)	0.005 (3)

### Geometric parameters (Å, º)

**Table:**

N1—C1	1.315 (5)	C7—H7	0.9300
N1—H1A	0.8600	C8—C9	1.506 (5)
N1—H1B	0.8600	C8—H8A	0.9700
N2—C7	1.348 (4)	C8—H8B	0.9700
N2—C5	1.367 (4)	C9—C10	1.384 (5)
N2—C8	1.476 (4)	C9—C14	1.393 (5)
O1—C1	1.224 (4)	C10—C11	1.397 (5)
C1—C2	1.508 (5)	C10—H10	0.9300
C2—C7	1.363 (4)	C11—C12	1.385 (7)
C2—C3	1.390 (5)	C11—H11	0.9300
C3—C4	1.371 (5)	C12—C13	1.357 (7)
C3—H3	0.9300	C12—H12	0.9300
C4—C5	1.374 (5)	C13—C14	1.369 (6)
C4—H4	0.9300	C13—H13	0.9300
C5—C6	1.494 (4)	C14—C15	1.505 (6)
C6—H6A	0.9600	C15—H15A	0.9600
C6—H6B	0.9600	C15—H15B	0.9600
C6—H6C	0.9600	C15—H15C	0.9600
			
C1—N1—H1A	120.0	N2—C8—C9	113.5 (3)
C1—N1—H1B	120.0	N2—C8—H8A	108.9
H1A—N1—H1B	120.0	C9—C8—H8A	108.9
C7—N2—C5	121.3 (3)	N2—C8—H8B	108.9
C7—N2—C8	118.4 (3)	C9—C8—H8B	108.9
C5—N2—C8	120.3 (3)	H8A—C8—H8B	107.7
O1—C1—N1	123.6 (4)	C10—C9—C14	120.4 (3)
O1—C1—C2	118.9 (4)	C10—C9—C8	121.8 (3)
N1—C1—C2	117.5 (3)	C14—C9—C8	117.7 (3)
C7—C2—C3	118.1 (3)	C9—C10—C11	119.7 (4)
C7—C2—C1	117.9 (3)	C9—C10—H10	120.2
C3—C2—C1	124.0 (3)	C11—C10—H10	120.2
C4—C3—C2	119.6 (3)	C12—C11—C10	119.2 (4)
C4—C3—H3	120.2	C12—C11—H11	120.4
C2—C3—H3	120.2	C10—C11—H11	120.4
C3—C4—C5	121.4 (3)	C13—C12—C11	120.0 (4)
C3—C4—H4	119.3	C13—C12—H12	120.0
C5—C4—H4	119.3	C11—C12—H12	120.0
N2—C5—C4	117.8 (3)	C12—C13—C14	122.3 (5)
N2—C5—C6	120.4 (3)	C12—C13—H13	118.8
C4—C5—C6	121.8 (3)	C14—C13—H13	118.8
C5—C6—H6A	109.5	C13—C14—C9	118.4 (4)
C5—C6—H6B	109.5	C13—C14—C15	120.3 (4)
H6A—C6—H6B	109.5	C9—C14—C15	121.3 (4)
C5—C6—H6C	109.5	C14—C15—H15A	109.5
H6A—C6—H6C	109.5	C14—C15—H15B	109.5
H6B—C6—H6C	109.5	H15A—C15—H15B	109.5
N2—C7—C2	121.7 (3)	C14—C15—H15C	109.5
N2—C7—H7	119.1	H15A—C15—H15C	109.5
C2—C7—H7	119.1	H15B—C15—H15C	109.5
			
O1—C1—C2—C7	−5.5 (5)	C1—C2—C7—N2	−179.2 (3)
N1—C1—C2—C7	175.8 (3)	C7—N2—C8—C9	−103.9 (3)
O1—C1—C2—C3	174.2 (4)	C5—N2—C8—C9	74.9 (4)
N1—C1—C2—C3	−4.5 (5)	N2—C8—C9—C10	17.3 (4)
C7—C2—C3—C4	−1.4 (5)	N2—C8—C9—C14	−164.3 (3)
C1—C2—C3—C4	178.9 (3)	C14—C9—C10—C11	1.2 (5)
C2—C3—C4—C5	−0.1 (5)	C8—C9—C10—C11	179.6 (3)
C7—N2—C5—C4	−2.2 (4)	C9—C10—C11—C12	−0.9 (6)
C8—N2—C5—C4	179.0 (3)	C10—C11—C12—C13	0.4 (7)
C7—N2—C5—C6	177.6 (3)	C11—C12—C13—C14	−0.2 (7)
C8—N2—C5—C6	−1.1 (4)	C12—C13—C14—C9	0.5 (6)
C3—C4—C5—N2	1.9 (5)	C12—C13—C14—C15	179.6 (4)
C3—C4—C5—C6	−177.9 (3)	C10—C9—C14—C13	−1.0 (5)
C5—N2—C7—C2	0.8 (5)	C8—C9—C14—C13	−179.5 (3)
C8—N2—C7—C2	179.5 (3)	C10—C9—C14—C15	179.9 (4)
C3—C2—C7—N2	1.1 (5)	C8—C9—C14—C15	1.4 (5)

### Hydrogen-bond geometry (Å, º)

**Table:**

*D*—H···*A*	*D*—H	H···*A*	*D*···*A*	*D*—H···*A*
N1—H1*A*···Br1	0.86	2.67	3.477 (3)	157
N1—H1*B*···O1^i^	0.86	2.35	3.207 (4)	173
C3—H3···O1^i^	0.93	2.22	3.149 (5)	174
C4—H4···Br1^i^	0.93	2.78	3.712 (4)	175
C6—H6*C*···Br1^ii^	0.96	2.73	3.638 (3)	157
C8—H8*B*···Br1^iii^	0.97	2.87	3.782 (3)	156

Symmetry codes: (i) −*x*+1/2, *y*−1/2, −*z*+1/2; (ii) −*x*+1, *y*, −*z*+1/2; (iii) −*x*+1/2, *y*+1/2, −*z*+1/2.
